# Decoupling Electronic–Ionic Transport and Catalysis Enables High‐Performance, Chemically Stable Sr‐Free Air Electrodes for High‐Temperature Solid Oxide Cells

**DOI:** 10.1002/advs.77023

**Published:** 2026-08-05

**Authors:** Ji‐eun Won, Wooseok Lee, Jaehyun Seo, Dogeun Yoon, Ho‐Il Ji, HeeChan Kang, Jun Hyuk Kim, Hwitae Kim, Ji Wan Kim, Jongsup Hong, Kyung Joong Yoon

**Affiliations:** ^1^ Hydrogen Energy Materials Research Center Korea Institute of Science and Technology Seoul Republic of Korea; ^2^ School of Mechanical Engineering Yonsei University Seoul Republic of Korea; ^3^ Nanomaterials Science and Engineering Korea University of Science and Technology (UST) KIST Campus Seoul Republic of Korea; ^4^ Department of Future Energy Engineering Sungkyunkwan University Suwon Republic of Korea; ^5^ SKKU Institute of Energy Science and Technology (SIEST) Sungkyunkwan University Suwon Republic of Korea; ^6^ Materials Research & Engineering Center Hyundai Motor Company Uiwang‐si Gyeonggi‐do Republic of Korea

**Keywords:** chromium, degradation, perovskite, solid oxide cell, strontium

## Abstract

Although strontium is a major origin of various degradation mechanisms in high‐temperature electrochemical cells, it is inevitably employed as an A‐site dopant in perovskite air electrodes to ensure adequate functionality. Herein, we report a high‐performance Sr‐free air electrode achieved by independently tailoring electronic conduction, ionic transport, and surface catalytic activity. High electronic conductivity is realized using multi‐valent B‐site perovskites with fully La‐occupied A‐sites, while efficient ionic transport is provided by oxygen‐interstitial Ruddlesden–Popper phases without Sr doping. Moreover, highly active nanocatalysts are incorporated via infiltration to accelerate surface reaction kinetics, enabling electrochemical performance comparable to that of state‐of‐the‐art Sr‐containing electrodes. Full cells employing this electrode exhibit exceptional durability under harsh electrolysis conditions, particularly under severe Cr vapor exposure. While conventional Sr‐based electrodes exhibit rapid degradation of ∼15% within 100 h owing to reactions between segregated Sr and Cr vapor, the Sr‐free electrode maintains stable performance with no detectable decay over 200 h of continuous operation. This design strategy offers a scalable and immediately applicable pathway to resolve critical durability issues in high‐temperature electrochemical energy systems.

## Graphical abstract

A Sr‐free air electrode for high‐temperature solid oxide cells is designed by independently tailoring electronic conduction, ionic transport, and surface catalysis using complementary Sr‐free phases and infiltrated nanocatalysts. It delivers performance comparable to state‐of‐the‐art Sr‐containing electrodes while completely suppressing Cr‐induced degradation, maintaining stable operation over 200 h under harsh electrolysis conditions where conventional electrodes rapidly degrade.

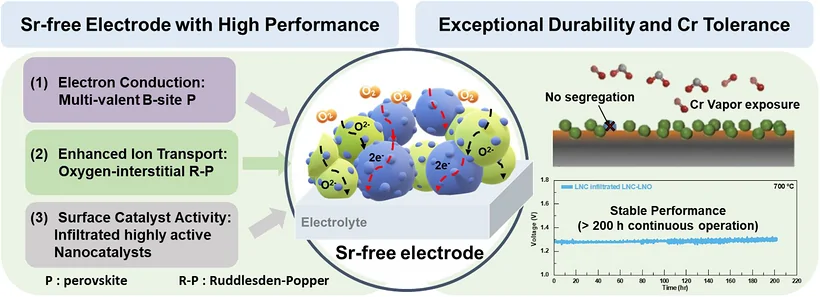

## 1 Introduction

Amid the growing demand for clean energy technologies, high‐temperature electrochemical devices based on solid oxides have emerged as a promising next‐generation energy platform. Operating typically above 700°C, solid oxide cells (SOCs) offer unique advantages, including exceptional energy conversion efficiency and multifunctionality [1, 2]. In particular, their importance has been further amplified by rapidly expanding sectors such as AI data centers and clean hydrogen production [3, 4]. However, commercialization and large‐scale deployment remain constrained by limited long‐term stability, as sustained high‐temperature operation induces complex physicochemical degradation that shortens device lifetime [5]. Overcoming high‐temperature degradation therefore represents the central challenge to realizing the full potential of SOC technology in emerging energy markets and meeting growing societal demands.

Among SOC components, air electrodes are particularly susceptible to severe degradation arising from coupled chemical and structural evolution and interactions with external contaminants [6, 7]. State‐of‐the‐art air electrodes are predominantly based on Sr‐containing perovskite oxides, featuring La‐based A‐sites partially substituted with Sr and B‐sites occupied by transition metals such as Mn, Co, and Fe, including (La,Sr)CoO_3‐δ_ (LSC), (La,Sr)(Co,Fe)O_3‐δ_ (LSCF), and (La,Sr)MnO_3‐δ_ (LSM) [8, 9]. In these materials, Sr is intrinsically unstable in the perovskite lattice and readily segregates to surfaces and interfaces, triggering multiple degradation pathways [10, 11]. Specifically, surface Sr segregation leads to the formation of SrO‐rich phases that degrade catalytic activity [12], while the segregated Sr readily reacts with airborne impurities, including Cr vapor, CO_2_, and H_2_O, to form detrimental secondary phases [13]. In addition, Sr migrates to the electrode–electrolyte interface and reacts with the yttria‐stabilized zirconia (YSZ) electrolyte, forming insulating SrZrO_3_ phases [14]. The continuous depletion of Sr ultimately disrupts lattice stoichiometry and drives chemical decomposition of the air electrode [15].

Because Sr is the primary origin of multiple degradation phenomena, its elimination from air electrodes has been pursued as the most straightforward mitigation strategy [12, 16]. However, Sr is also essential for achieving high electronic and ionic conductivity as well as strong catalytic activity [17], making it extremely challenging to attain adequate electrochemical performance in its absence. For instance, substitution of Sr with other divalent cations (e.g., Ca or Ba) can partially alleviate certain Sr‐related degradation phenomena [17, 18]; however, the accompanying penalties—including the loss of electrochemical performance and compromised structural and phase stability—generally outweigh the benefits. Alternatively, Sr can be eliminated from the A‐site by adopting fully trivalent A‐site perovskites without acceptor doping and employing mixed B‐site elements, such as La(Co,Fe)O_3‐δ_ and La(Ni,Fe)O_3‐δ_ [19, 20]; however, the lack of A‐site acceptor doping restricts oxygen vacancy formation, resulting in low oxygen‐ion conductivity [21]. Alternative crystal structures, such as Ruddlesden–Popper phases, intrinsically avoid Sr incorporation [22], and certain compositions, including La_2_NiO_4+δ_ and Pr_2_NiO_4+δ_, exhibit high oxygen‐ion conductivity enabled by an interstitial oxygen transport mechanism [23]; however, their relatively low electronic conductivity and limited phase stability under processing conditions remain significant challenges [24]. In summary, although the promising performance of several candidate Sr‐free electrode materials has been demonstrated recently [25, 26, 27, 28, 29, 30], achieving a balanced combination of the desired electrode properties without Sr in realistic devices remains highly challenging.

To overcome these fundamental limitations, this study adopts a decoupled design strategy for Sr‐free air electrodes, in which electronic conductivity, ionic conductivity, and catalytic activity are independently optimized by integrating Sr‐free materials with complementary properties. By constructing a composite electrode with surface‐engineered nanocatalysts, the Sr‐free electrode achieves electrochemical performance comparable to that of state‐of‐the‐art Sr‐containing electrodes, while exhibiting markedly enhanced chemical stability under harsh operating conditions. The contributions of individual constituent phases to the overall electrochemical performance and long‐term durability are systematically investigated through combined electrochemical evaluation, theoretical modeling, and post‐mortem characterization.

## 2 Results and Discussion

### 2.1 Rationale for Designing High‐Performance Sr‐Free Air Electrodes

In the air electrode, the oxygen exchange reaction involves the transfer of oxide ions and electrons (Equation 1), and efficient electrode operation requires effective transport of both charge carriers together with fast surface exchange kinetics.

(1) $$O_{2\left(g\right)}+4e^{-}\;↔2O^{2-}\;$$

State‐of‐the‐art Sr‐containing mixed ionic‐ and electronic‐conductors (MIECs), such as LSC and LSCF, intrinsically fulfill these requirements within a single phase and can function as standalone electrodes providing efficient electronic transport, ionic transport, and surface catalytic activity (Figure 1a). In the absence of Sr, however, achieving all of these functionalities within a single phase becomes inherently challenging. Here, we address this limitation by decoupling and independently engineering each functionality using complementary Sr‐free components. Figure 1b schematically illustrates the Sr‐free composite electrode architecture, in which percolating electronic and ionic conduction pathways are integrated, and nanocatalysts are deposited on the internal surfaces. In the composite, La(Ni,Co)O_3−δ_ (LNC) and La_2_NiO_4+δ_ (LNO) are employed to provide efficient electronic and oxygen‐ion conduction, respectively. LNC exhibits high p‐type electronic conductivity [31], reaching ∼1000 S cm^−1^ at 700°C (Figure S1), originating from the mixed‐valence states of Ni and Co that enable efficient hole transport via strong B–O–B orbital overlap [32, 33]. However, oxygen‐ion transport in LNC is limited because charge compensation predominantly proceeds via B‐site redox rather than oxygen‐vacancy formation, resulting in a low oxygen vacancy concentration and high migration barriers [34]. This drawback is compensated by LNO, which features a layered Ruddlesden–Popper structure capable of accommodating a high concentration of interstitial oxygen ions, thereby enabling rapid oxygen‐ion transport [35]. In contrast, LNO exhibits relatively low electronic conductivity (∼150 S cm^−1^ at 700°C (Figure S1)) owing to the absence of aliovalent A‐site acceptor doping [36, 37]. By combining LNC and LNO, the composite electrode compensates for the intrinsic limitations of each individual phase and establishes continuous conduction pathways for both electrons and oxygen ions.

**FIGURE 1:**
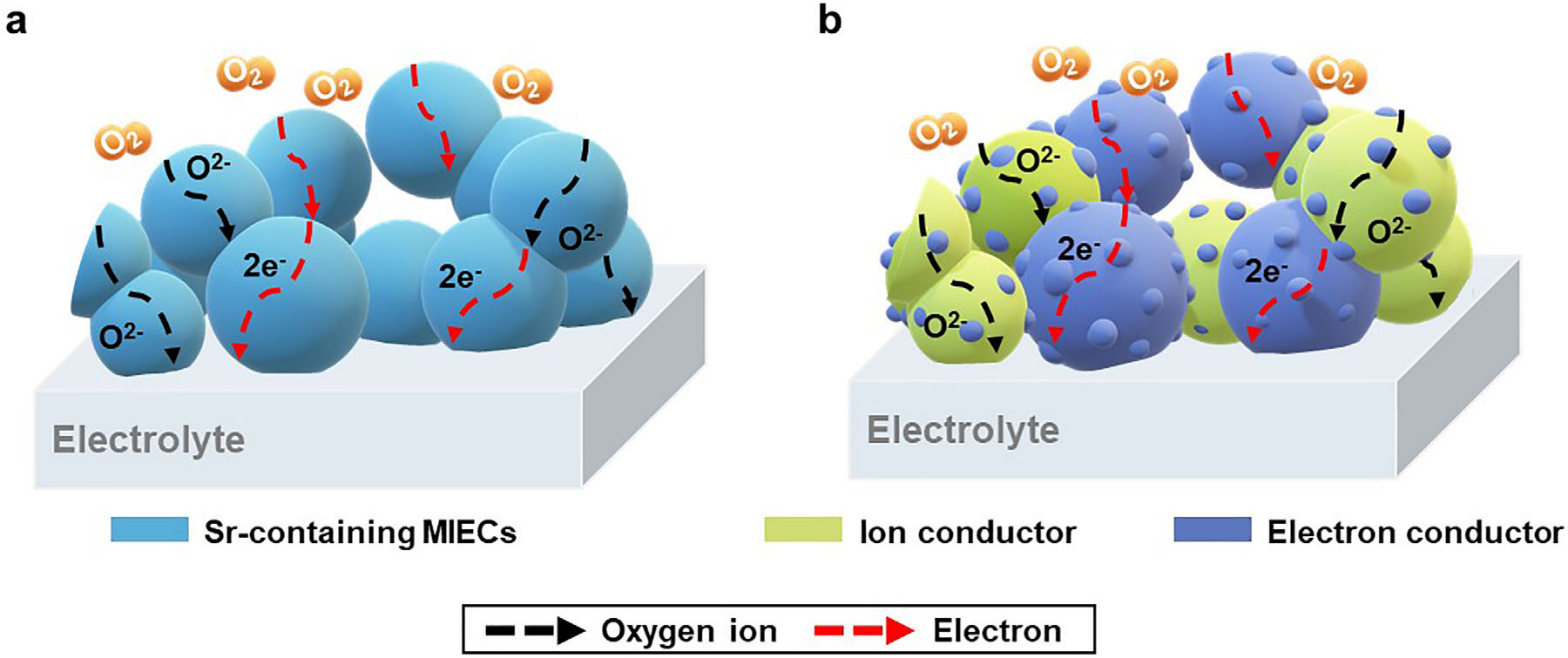
Schematic illustrations of electrode architectures. (a) Conventional Sr‐containing MIEC electrode, in which electronic conduction, oxygen‐ion transport, and catalytic activity are provided by a single material. (b) Sr‐free composite electrode architecture, in which percolating electronic‐ and ionic‐conducting phases are integrated, and nanocatalysts are deposited on the internal surfaces.

To enhance surface reaction kinetics, nanocatalysts were introduced onto the internal surfaces of the porous electrode via an infiltration process, in which a precursor solution is injected into the pore network and subsequently converted into nanoparticles through thermal treatment [38]. In conventional infiltration approaches, precise control over phase purity, particle morphology, and spatial distribution remains challenging, as liquid migration within porous scaffolds is difficult to regulate and often accompanied by uncontrolled cation redistribution. In our previous work, we addressed these limitations by regulating complexation and precipitation behavior, thereby enabling the formation of uniformly distributed, phase‐pure perovskite nanoparticles [39, 40]. Here, we apply this process to introduce Sr‐free LNC nanocatalysts into the LNC–LNO composite electrode, thereby further enhancing surface catalytic activity and fulfilling all key requirements for high electrode performance. Notably, the constituent materials were selected to have closely matched chemical compositions to ensure thermochemical compatibility at high temperatures.

### 2.2 Fabrication and Evaluation of Sr‐Free Composite Electrode

For the implementation of new electrode materials, their thermochemical compatibility with adjacent cell components must be ensured to prevent detrimental chemical reactions, and suitable processing conditions should be identified accordingly. In standard cell architectures, the LNC–LNO composite is deposited on a GDC interlayer, necessitating chemical compatibility among LNC, LNO, and GDC. Our previous study showed that LNO reacts with GDC above 1000°C: La in La_2_NiO_4_ diffuses into the GDC lattice, and this loss of La shifts the residual nickelate toward a more Ni‐rich composition, driving the formation of higher‐order Ruddlesden–Popper phases of the homologous series La_n+1_Ni_n_O_3n+1_, such as La_3_Ni_2_O_7_ and La_4_Ni_3_O_10_ [41]. Since the interstitial‐oxygen content and transport properties as well as the electronic conductivity depend strongly on the Ruddlesden–Popper order, such chemical interaction makes the key electrode properties difficult to control and reproduce. Accordingly, the processing temperature should not exceed 1000°C. The chemical compatibility among the three material pairs, LNO–GDC, LNC–GDC, and LNO–LNC, was examined at 950°C using XRD analysis (Figure 2a–c). No noticeable chemical interactions were detected for any of the pairs, indicating that 950°C provides an appropriate processing window without inducing detrimental interfacial reactions.

**FIGURE 2:**
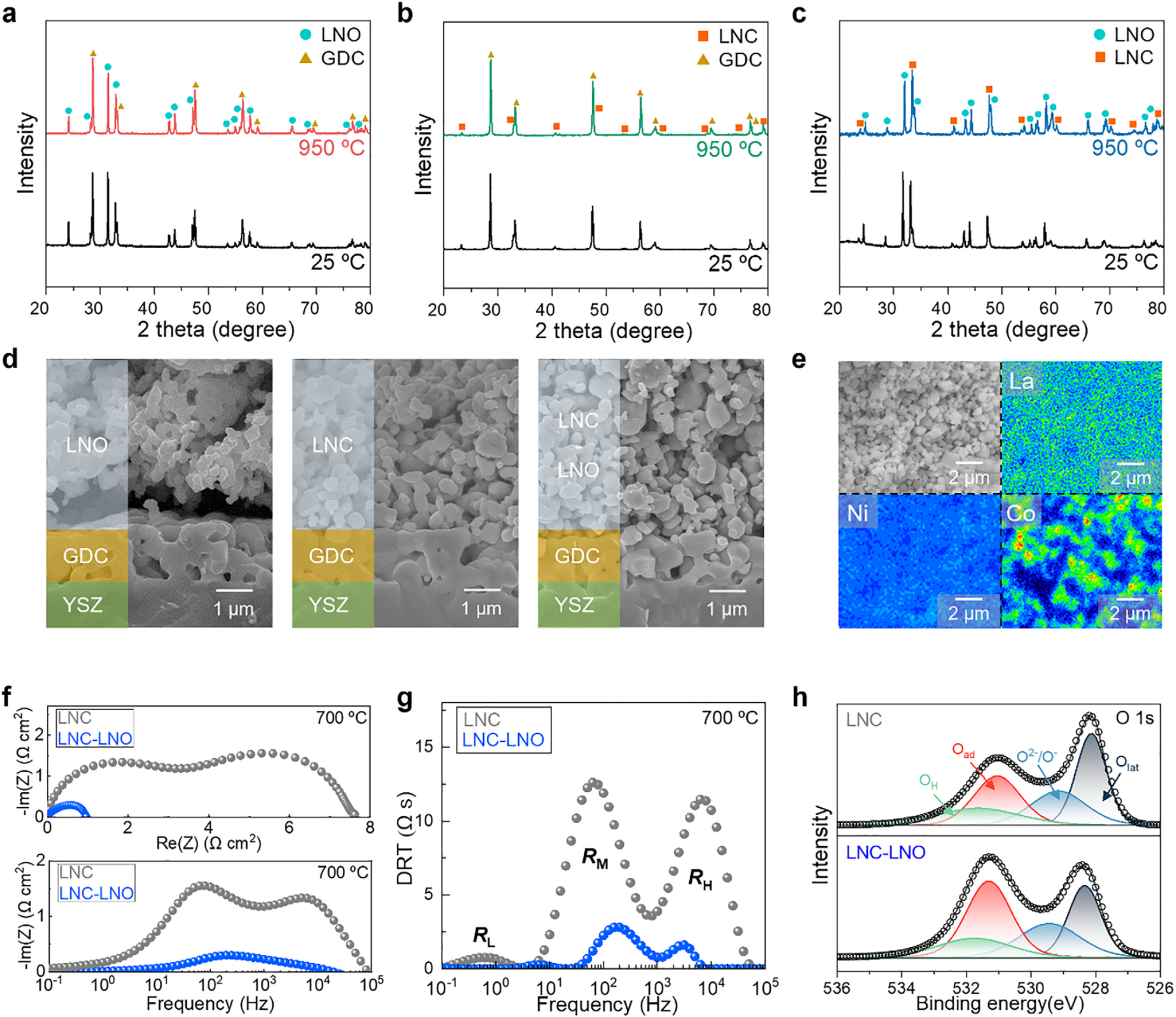
Fabrication and electrochemical evaluation of the Sr‐free composite electrode. (a–c) XRD patterns of (a) LNO–GDC, (b) LNC–GDC, and (c) LNO–LNC mixtures measured before and after heat treatment at 950°C. (d) Cross‐sectional SEM images showing the interfaces between LNO (left), LNC (middle), and LNC–LNO composite (right) electrodes deposited on the GDC substrate. (e) SEM image and corresponding EPMA elemental maps of the LNC–LNO composite electrode. (f) Nyquist (upper) and Bode (lower) plots of the impedance spectra of symmetric cells with LNC and LNC–LNO composite electrodes measured at 700°C. (g) DRT analysis of the impedance spectra at 700°C. (h) O 1s XPS spectra of LNC and LNC–LNO composite electrodes with peak‐fitting results.

However, when sintered at 950°C, the single‐phase LNO layer exhibits poor interfacial adhesion to the underlying GDC layer, resulting in partial interfacial delamination (Figure 2d, left), consistent with previous reports [24, 41]. In contrast, a single‐phase LNC layer forms a well‐adhered interface with GDC under identical conditions (Figure 2d, middle). In the LNC–LNO composite electrode, the LNC particles serve as a bonding framework that establishes strong adhesion to the underlying GDC layer and binds the poorly sintering LNO network together. Consequently, the composite electrode develops a mechanically robust, intact interface with GDC after sintering at 950°C (Figure 2d, right)—that is, within the temperature window where no LNO–GDC reaction occurs—thereby establishing reliable physical bonding to the GDC interlayer while preserving the chemical integrity of both phases. The SEM image and EPMA elemental maps of the LNC–LNO composite electrode in Figure 2e reveal a uniform distribution of LNO and LNC particles with an average size of ∼500 nm. The homogeneous mixing of the two phases is clearly evidenced by the Co elemental map, as Co is the only element that differentiates LNC from LNO.

Electrochemical performance was evaluated using symmetric cells, in which a ∼6 µm‐thick composite electrode was applied on both sides of a ∼3 mm‐thick GDC substrate and covered with a ∼10 µm‐thick LNC current‐collecting layer (Figure S2). The LNC–LNO composite electrode serves as the functional layer, where the oxygen reduction/evolution reactions take place, enabling rapid electronic and ionic transport and providing catalytically active surface sites. The outer current‐collecting layer minimizes contact resistance and facilitates lateral current distribution, for which single‐phase LNC is well suited owing to its high electronic conductivity. For comparison, symmetric cells with single‐phase LNC electrodes were also fabricated and tested (Figure S3). In the Nyquist plot measured at 700°C (Figure 2f, upper), the single‐phase LNC electrode exhibits a large polarization resistance of 7.62 Ω cm^2^, whereas incorporation of LNO reduces the polarization resistance to 0.97 Ω cm^2^, demonstrating that LNO effectively compensates for the limited ionic conductivity of LNC and substantially enhances the overall electrode performance. The Bode plot of the imaginary impedance (Figure 2f, lower) further shows that introducing LNO into LNC markedly improves the impedance response over the entire frequency range. In composite electrodes, the relative fraction of the two constituent phases is a critical parameter governing the performance. Accordingly, we compared the impedance spectra of LNC–LNO composite electrodes with different LNC:LNO weight ratios (60:40, 50:50, and 40:60); the 50:50 electrode exhibited the lowest polarization resistance, confirming that a balanced phase ratio provides favorable electrochemical performance (Figure S4). The impedance spectra were further analyzed using distribution of relaxation times (DRT) analysis, which enables high‐resolution deconvolution of electrochemical processes through mathematical calculations [42]. In Figure 2g, three characteristic contributions were identified at high‐ (10^3^–10^5^ Hz), mid‐ (10^1^–10^3^ Hz) and low‐ (10^−^^1^–10^0^ Hz) frequency ranges for both the LNC and LNC–LNO composite electrodes. To systematically identify these processes, we performed *pO_2_*‐dependent impedance measurements on an LNC–LNO composite electrode, varying *pO_2_* from 0.10 to 1.00 atm (Figure S5). The total polarization resistance increased with decreasing *pO_2_* (Figure S5a). Under all *pO_2_* conditions, the DRT analysis identified three rate‐limiting processes (Figure S5b). Based on these DRT results, the impedance spectra were fitted to an equivalent‐circuit model composed of three *R–CPE* elements, and the resistances of the individual contributions were quantitatively obtained (Figure S5c). The reaction order for each resistance component was determined from its *pO_2_* dependence using the relationship *R = k·(pO_2_)^−n^* (Figure S5d) [43]. The high‐frequency resistance exhibited a reaction order of *n* = 0.24, close to 1/4, indicating that this process is mainly associated with charge transfer and oxygen‐incorporation reactions [44, 45]. The mid‐frequency resistance showed *n* = 0.35, close to 3/8, suggesting that this process is associated with electron transfer to adsorbed oxygen species during the oxygen surface exchange process [46, 47]. The high‐ and mid‐frequency arcs account for the major portion of the overall polarization resistance and exhibit a monotonic increase in reaction order with decreasing characteristic frequency [43, 48]. In contrast, the low‐frequency contribution was considerably smaller than the high‐ and mid‐frequency contributions, accounting for less than 7% of the total polarization resistance, and was largely embedded within the mid‐frequency response. Therefore, this minor low‐frequency feature was excluded from the quantitative *pO_2_*‐dependence analysis. For the single‐phase LNC electrode, both the high‐ and mid‐frequency contributions are dominant, reflecting severe limitations in charge transport and surface exchange arising from poor oxygen‐ion conductivity. These contributions are markedly reduced in the LNC–LNO composite electrode, confirming the beneficial role of LNO in enhancing ionic transport and surface reaction kinetics. X‐ray photoelectron spectroscopy (XPS) analysis further supports the improved surface reaction kinetics in the composite electrode (Figure 2h). In the O 1s spectra, the ratio of adsorbed oxygen to lattice oxygen (O_ad_/O_lat_) increases from 0.83 for the LNC electrode to 1.45 for the LNC–LNO composite (Table S1), indicating a higher concentration of oxygen vacancies and accelerated oxygen exchange kinetics [49]. Nevertheless, the mid‐frequency contribution in the DRT analysis remains dominant compared with typical MIEC air electrodes [40], indicating that further enhancement of surface reaction kinetics is required.

### 2.3 Nanocatalyst Infiltration to Enhance Surface Reaction Kinetics

To further enhance surface oxygen exchange kinetics, LNC nanoparticles were introduced onto the porous LNC–LNO composite electrode via infiltration, enabling uniform dispersion of nanoscale catalysts throughout the internal electrode surface. LNC was selected as the nanocatalyst because it is one of the constituent phases of the composite, ensuring chemical compatibility and avoiding interfacial reactions or secondary phase formation. To determine the optimum composition of nanocatalysts, La(Ni_1‐x_Co_x_)O_3‐δ_ (LNC) nanoparticles with varying Co contents (0.3 ≤ x ≤ 0.6) were synthesized via a urea–glycine complexation–based infiltration process [50]. XRD analysis in Figure 3a shows the formation of single‐phase perovskite structures at low Co contents, whereas increasing Co contents above 50% induces peak splitting, possibly associated with local distortion of the BO_6_ octahedra [51]. SEM analysis (Figure 3b and Figure S6) reveals that the infiltrated LNC nanoparticles are uniformly distributed over the internal surfaces of the porous LNC–LNO composite electrode, regardless of nanoparticle composition. Furthermore, scanning transmission electron microscopy–energy‐dispersive x‐ray spectroscopy (STEM–EDS) analysis in Figure 3c shows an average particle size of ∼50 nm and a homogeneous distribution of constituent elements within the nanoparticles.

**FIGURE 3:**
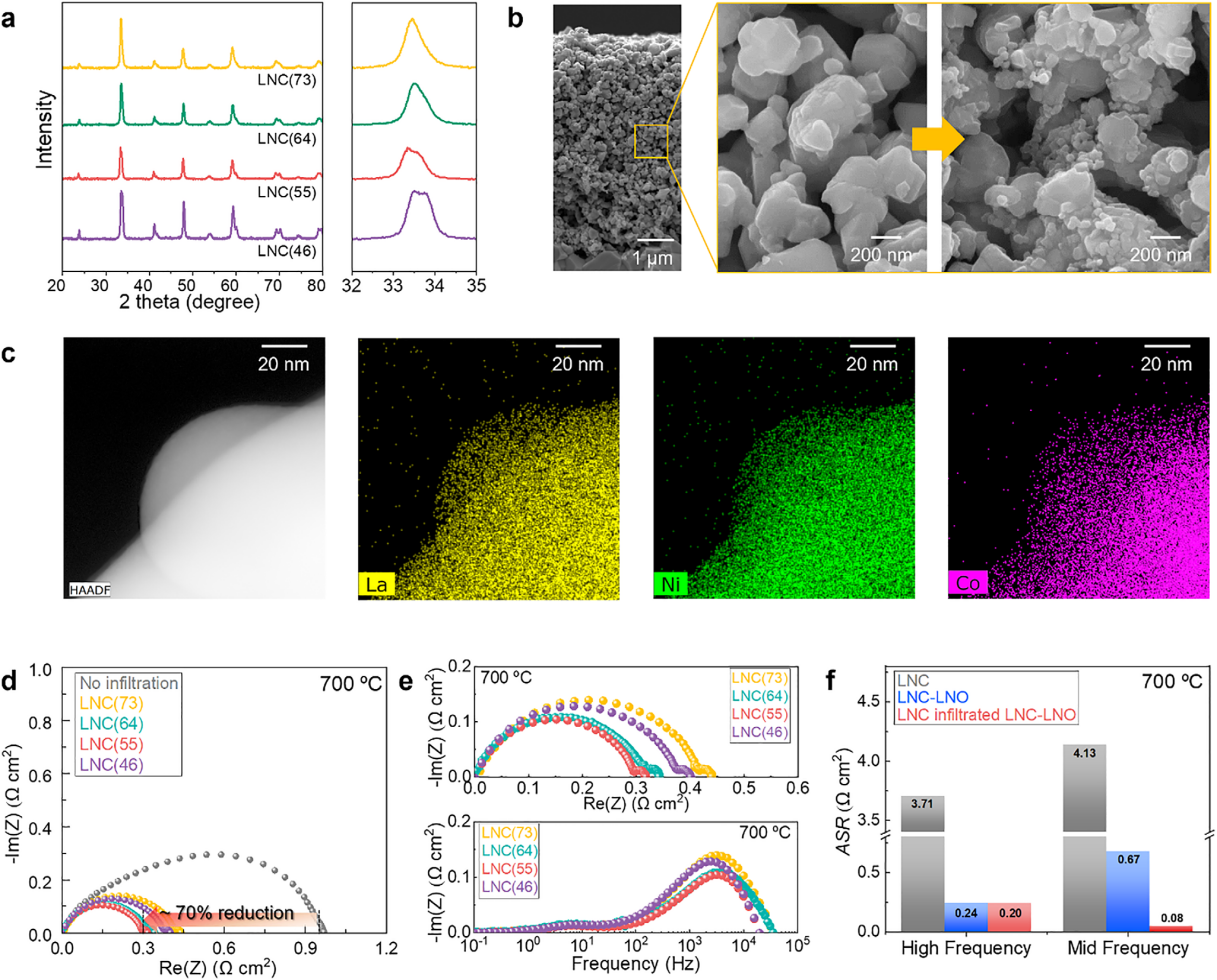
LNC nanocatalyst infiltration into the LNC–LNO composite electrode. (a) XRD patterns of La(Ni_1‐x_Co_x_)O_3‐δ_ nanoparticles with varying Ni and Co contents (0.3 ≤ x ≤ 0.6) synthesized from infiltration solution. LNC(a,b) denotes La(Ni_a_Co_b_)O_3−δ_, where a and b represent the molar fractions of Ni and Co. (b) SEM images of the LNC–LNO composite electrode (left), internal surface before infiltration (middle), and after infiltration of La(Ni_0.5_Co_0.5_)O_3‐δ_ nanocatalysts (right). (c) STEM–EDS analysis of infiltrated LNC nanoparticles. (d) Impedance spectra of LNC–LNO composite electrodes infiltrated with different LNC nanocatalyst compositions, compared with the pristine electrode. (e) Comparison of impedance spectra for electrodes infiltrated with various LNC compositions. (f) Comparison of high‐ and mid‐frequency impedance contributions of single‐phase LNC, LNC–LNO composite, and LNC–infiltrated LNC–LNO electrodes.

The electrochemical impact of LNC nanocatalyst infiltration was evaluated using symmetric cells at 700°C. Compared with the catalyst‐free LNC–LNO composite electrode, all infiltrated electrodes exhibited a pronounced reduction in polarization resistance of approximately 55–70% (Figure 3d). Performance improved with increasing Co content up to 50% but deteriorated at higher levels, with La(Ni_0.5_Co_0.5_)O_3‐δ_ delivering the lowest polarization resistance of 0.318 Ω cm^2^ (Figure 3e). The activity enhancement with increasing Co content originates from the favorable electronic and redox characteristics of Co in the perovskite B‐site. Compared with Ni, Co exhibits more accessible Co^3+^/Co^4+^ redox couples under typical SOC operating conditions, which facilitate the charge‐transfer steps of the oxygen reduction/evolution reaction. In addition, the higher 3d‐electron count of Co generates a Co 3d–O 2p band more strongly hybridized near the Fermi level than that of Ni, lowering the oxygen vacancy formation energy and enhancing surface oxygen exchange kinetics [52, 53]. However, the activity gain saturates and then reverses beyond x = 0.5. This is attributable to a deterioration of structural integrity at high Co content, manifested by the clear peak splitting observed in the XRD pattern of La(Ni_0.4_Co_0.6_)O_3‐δ_ (Figure 3a). The splitting is associated with local distortion of the BO_6_ octahedra, which is well documented in Co‐rich LaCoO_3_‐based perovskites as a structural instability driven by the Jahn–Teller‐active intermediate‐spin Co^3+^ state and by Co^3+^/Co^4+^ disproportionation at elevated temperatures [51, 54]. Such local distortions disrupt the long‐range B–O–B connectivity that mediates electron transport and oxygen‐ion exchange, thereby degrading catalytic activity despite the higher Co fraction nominally being beneficial. The impedance spectra of single‐phase LNC, LNC–LNO composite, and LNC–infiltrated LNC–LNO electrodes were compared using deconvoluted high‐ and mid‐frequency impedance obtained by DRT analysis and equivalent circuit fitting (Figure 3f). The single‐phase LNC electrode exhibits large high‐ and mid‐frequency impedances. Incorporation of LNO significantly reduces both contributions by providing effective oxygen‐ion conduction pathways; however, the mid‐frequency impedance remains dominant, suggesting sluggish surface reaction kinetics. This limitation is effectively addressed by LNC nanocatalyst infiltration, leading to a substantial reduction in the mid‐frequency impedance and overall enhancement of electrode performance.

### 2.4 Electrochemical Performance of Sr‐Free Air Electrodes in Full Cells

The electrochemical performance of Sr‐free air electrodes in full cells was evaluated under realistic operating conditions. While symmetric‐cell measurements are useful for isolating electrode electrochemical activity from many additional variables, full‐cell testing provides a more comprehensive assessment of practical applicability by incorporating additional factors, including electrical resistance, manufacturability, and thermochemical and thermomechanical compatibility. Three types of air electrodes—single‐phase LNC, LNC–LNO composite, and LNC–LNO composite infiltrated with LNC nanocatalysts—were fabricated and systematically investigated. The composition of the LNC nanocatalysts was La(Ni_0.5_Co_0.5_)O_3−δ_, which was identified as optimal in symmetric‐cell evaluations. The left panel of Figure 4a shows an SEM image of the full cell, consisting of a ∼400 µm‐thick Ni–YSZ fuel‐electrode‐support, a ∼10 µm‐thick functional layer, a ∼3 µm‐thick YSZ electrolyte, and a ∼2 µm‐thick GDC interlayer, onto which the air electrodes were deposited. The LNC–LNO composite electrode has a thickness of ∼20 µm, and a ∼15 µm‐thick LNC current‐collecting layer was applied on top (Figure 4a, middle). After infiltration, LNC nanocatalysts are uniformly distributed on the internal surfaces of the LNC–LNO composite electrode (Figure 4a, right). The single‐phase LNC electrode is ∼35 µm thick (Figure S7), which is comparable to the total thickness of the LNC–LNO composite electrode with the LNC current‐collecting layer, ensuring a fair comparison among the different electrode architectures.

**FIGURE 4:**
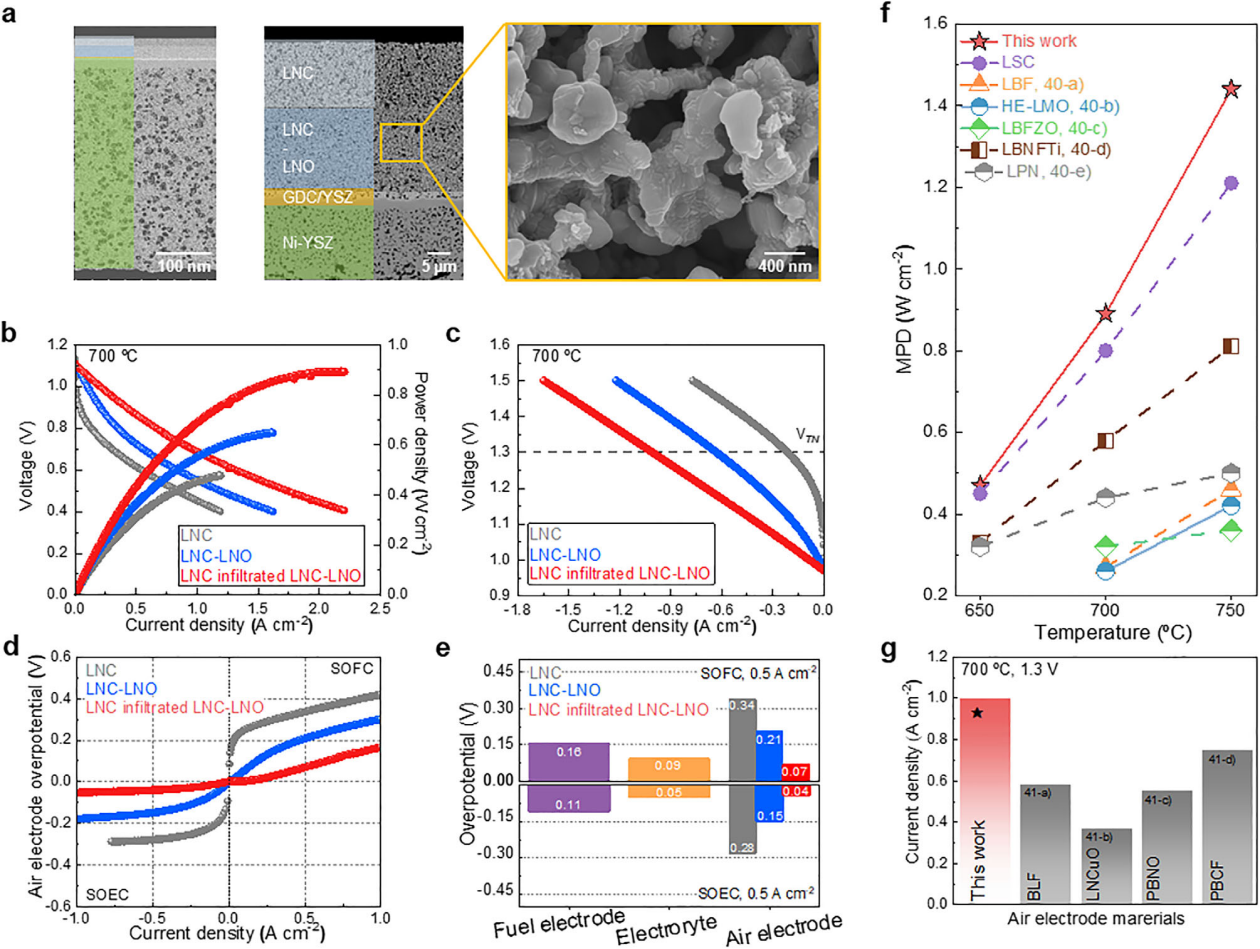
Evaluation of electrochemical performance of Sr‐free air electrodes in full cells. (a) Cross‐sectional SEM images of the fuel‐electrode‐supported cell with the LNC–LNO composite electrode and LNC current‐collecting layer (left and middle), and a magnified SEM image showing infiltrated LNC nanocatalysts uniformly distributed on the internal surfaces of the porous scaffold (right). (b) *I–V* curves and corresponding power density characteristics measured in fuel cell mode at 700°C for cells employing single‐phase LNC, LNC–LNO composite, and LNC–infiltrated LNC–LNO air electrodes. (c) *I–V* characteristics measured in electrolysis mode at 700°C for cells employing single‐phase LNC, LNC–LNO composite, and LNC–infiltrated LNC–LNO air electrodes. (d) Air‐electrode overpotential as a function of current density calculated using a multiphysics model. (e) Comparison of air electrode, electrolyte, and fuel‐electrode overpotentials at 0.5 A cm^−^^2^ calculated using the multiphysics model. (f,g) Comparison of f) maximum power densities in fuel cell mode and g) current densities at the thermoneutral voltage for the present work and previously reported Sr‐free air electrodes in the literature. (LBF: LaBa_0.5_Fe_0.5_O_3‐δ_; [59] HE‐LMO: LaMn_0.2_Fe_0.2_Co_0.2_Ni_0.2_Cu_0.2_O_3‐δ_; [60] LBFZO: La_0.5_Ba_0.5_Fe_0.95_Zr_0.05_O_3‐δ_; [61] LBNFTi: La_0.6_Ba_0.4_Ni_0.2_Fe_0.7_Ti_0.1_O_3‐δ_; [62] LPN: LaPrNiO_4+δ_; [63] BLF: Ba_0.95_La_0.05_FeO_3‐δ_; [64] LNCuO: La_2_Ni_0.8_Cu_0.2_O_4_; [65] PBNO: Pr_1.8_Ba_0.2_NiO_4+δ_; [66] PBCF: Pr_0.97_Ba_0.97_Co_1.5_Fe_0.5_O_5+δ_[67]).

The fabricated cells were tested using a standardized setup designed to reproduce a realistic stack environment, consisting of a metallic interconnect with standard channel/rib geometry, a glass‐ceramic sealant, and current‐collecting foam/mesh components (Figure S8). The I–V curves and corresponding power density characteristics measured in fuel cell mode at 700°C are shown in Figure 4b. The cell employing a single‐phase LNC air electrode exhibits a maximum power density of 0.50 W cm^−^^2^. Incorporation of the LNC–LNO composite improves the performance to 0.65 W cm^−^^2^, while subsequent LNC nanocatalyst infiltration further enhances it to 0.90 W cm^−^^2^. The beneficial effects of composite formation and nanocatalyst infiltration are particularly evident in the low‐current‐density region near open‐circuit conditions in the *I–V* curves. In this region, the cell with the single‐phase LNC air electrode exhibits a sharp voltage drop, indicative of large activation polarization arising from insufficient ionic transport in the predominantly electronic‐conducting LNC phase. The introduction of LNO effectively mitigates the initial voltage loss by providing continuous ionic conduction pathways, while the infiltration of LNC nanocatalysts further enhances surface catalytic activity, nearly eliminating the low‐current voltage drop and thereby substantially improving electrochemical performance. Consistent trends are observed in electrolysis mode (Figure 4c). The current density at the thermoneutral voltage increases from 0.22 A cm^−^^2^ for the single‐phase LNC electrode to 0.63 A cm^−^^2^ upon LNO incorporation and further to 1.01 A cm^−^^2^ after LNC nanocatalyst infiltration. The stepwise reduction in activation polarization is again clearly manifested in the low‐current‐density region in the I–V curves, where the abrupt voltage rise observed for the single‐phase LNC electrode is substantially suppressed by composite formation and nearly eliminated by nanocatalyst infiltration.

To gain a more comprehensive understanding of the electrochemical characteristics, the measured *I–V* curves were analyzed using a multiphysics simulation model. Using our previously reported model [55], we quantified the contributions of individual components to the overall cell performance, with particular focus on the air electrode. As shown in Figure 4d, the air electrode overpotential decreases markedly when the electrode evolves from the LNC single phase to the LNC–LNO composite and further to the LNC‐infiltrated LNC–LNO structure in both fuel cell and electrolysis modes. While the air electrode dominates the overall cell polarization in the LNC single‐phase electrode, this contribution is significantly reduced by introducing the LNC–LNO composite and further minimized through LNC infiltration, ultimately becoming lower than those of the fuel electrode and electrolyte in the LNC‐infiltrated LNC–LNO electrode (Figure 4e). Consequently, the LNC‐infiltrated LNC–LNO electrode achieves performance comparable to or slightly better than that of state‐of‐the‐art Sr‐containing air electrodes, such as LSC‐based electrodes (Figure S9), while also falling within the upper range of performance benchmarks reported by leading cell manufacturers [56, 57, 58]. It also represents one of the highest performances reported to date among Sr‐free air electrode systems in both fuel cell (Figure 4f and Table S2) [59, 60, 61, 62, 63] and electrolysis (Figure 4g and Table S3) [64, 65, 66, 67] modes. Collectively, these results demonstrate that independently regulating bulk ionic/electronic transport and surface catalytic activity enables high‐performance air electrodes without relying on Sr.

### 2.5 Long‐Term Stability Tests and Degradation Mechanism Investigation

To verify the mitigation of Sr‐induced degradation, long‐term durability tests and post‐operation analyses were performed. The long‐term tests were performed in electrolysis mode, which is known to impose more severe degradation than fuel cell mode [68, 69], at 700°C under a constant current density of 1.0 A cm^−^^2^ (Figure 5a). A cell employing the LNC–infiltrated LNC–LNO air electrode was tested and directly compared with a reference cell with a state‐of‐the‐art LSC air electrode. The stability test was performed using a metallic interconnect without a protective coating to evaluate the durability of the air electrode under harsh operating conditions involving severe exposure to Cr vapor. Both cells exhibited similar initial voltages, indicating comparable starting performance. However, during constant‐current operation, their degradation behaviors differed markedly. The LSC‐based cell exhibited rapid performance degradation from the early stage of operation, with the voltage increasing from approximately 1.3 to 1.5 V within 100 h. In contrast, the cell with the LNC–infiltrated LNC–LNO air electrode maintained a nearly constant voltage of ∼1.3 V over 200 h, demonstrating excellent operational stability with negligible degradation. Consistently, the *I–V* curves of this cell are nearly identical before and after the long‐term test (Figure 5b). We also examined the stability under fuel‐cell‐mode operation. As shown in Figure S10, the cell with the LNC‐infiltrated LNC–LNO air electrode maintained a nearly constant voltage under constant‐current operation at 0.5 A cm^−^^2^ for 100 h, whereas the cell with the LSC air electrode exhibited rapid voltage decay under the same conditions. These results confirm the exceptional stability of the Sr‐free LNC–infiltrated LNC–LNO air electrode, which exhibits durability far superior to that of the state‐of‐the‐art LSC electrode while maintaining comparable electrochemical performance.

**FIGURE 5:**
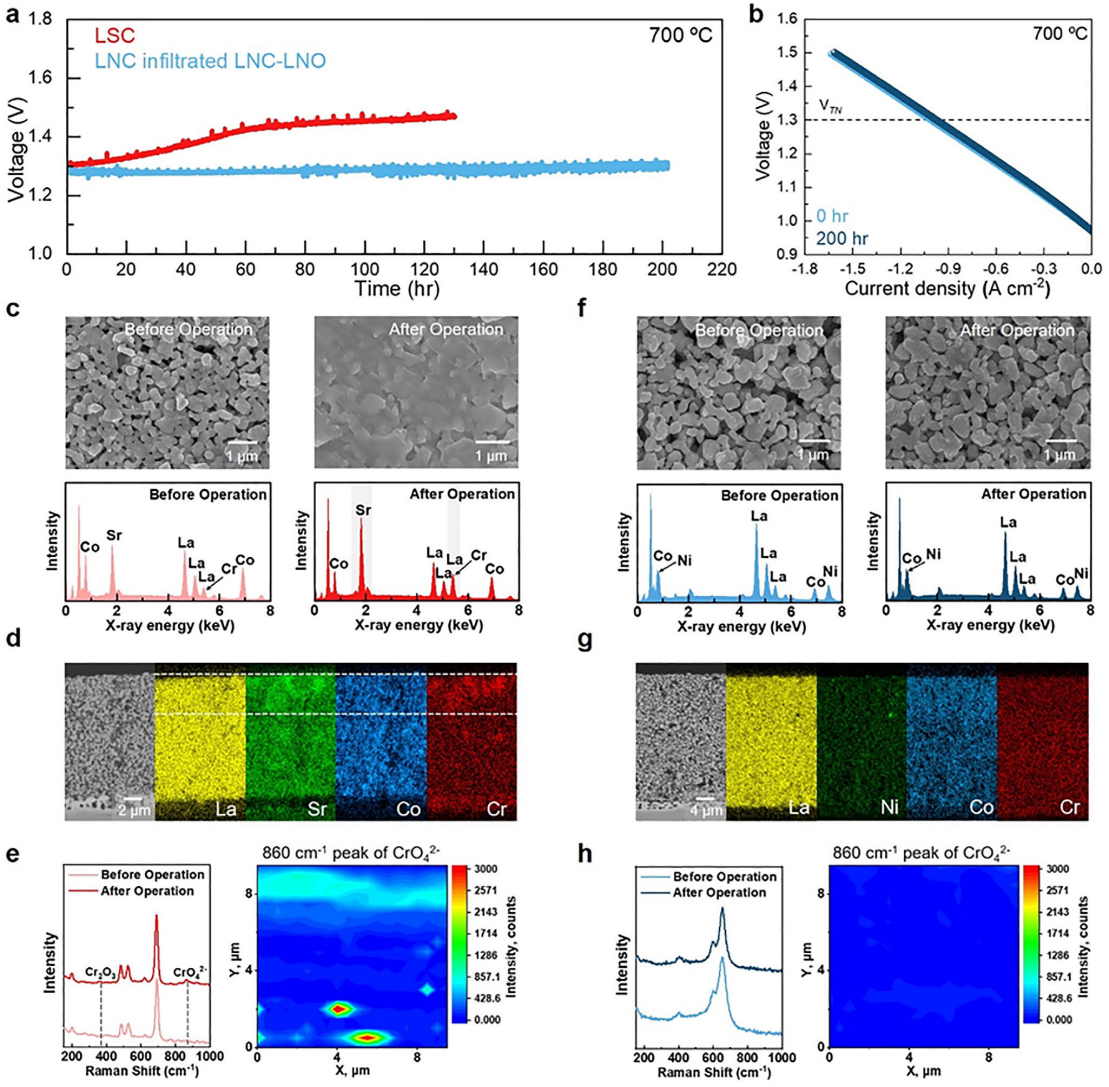
Long‐term durability tests and post‐mortem microstructural analysis of LSC and LNC–infiltrated LNC–LNO air electrodes. (a) Cell voltage as a function of time at a constant current density of 1.0 A cm^−^^2^ in electrolysis mode. (b) *I–V* characteristics of the cell with the LNC–infiltrated LNC–LNO air electrode before and after 200 h of operation. (c) Top‐view SEM images and EDS spectra of the LSC air electrode before and after the stability test. (d) Cross‐sectional SEM image and corresponding EDS elemental maps of the LSC air electrode after operation. (e) Raman spectra of the LSC air electrode before and after long‐term operation and Raman mapping after long‐term operation. (f) Top‐view SEM images and EDS spectra of the LNC–infiltrated LNC–LNO air electrode before and after the stability test. (g) Cross‐sectional SEM image and corresponding EDS elemental maps of the LNC–infiltrated LNC–LNO air electrode after operation. (h) Raman spectra of the LNC–infiltrated LNC–LNO air electrode before and after long‐term operation and Raman mapping after long‐term operation.

Post‐mortem analysis reveals the origin of the markedly different long‐term behaviors of the two cells. For the LSC electrode, SEM images of the top surface show pronounced structural alteration after long‐term operation (Figure 5c). Before testing, the electrode exhibits a uniform porous structure with particle sizes of 300–500 nm. After long‐term operation, however, localized surface densification is observed, accompanied by the formation of faceted particles that are distinctly different from the initially rounded morphology. EDS analysis reveals pronounced Sr and Cr signals after operation, indicating significant surface compositional changes. Cross‐sectional SEM images of the LSC electrode reveal the formation of a ∼0.5 µm‐thick dense layer on the outer surface (Figure S11), and the corresponding EDS mapping confirms pronounced Sr and Cr enrichment in this surface region (Figure 5d). Raman spectroscopy performed on the electrode surface (Figure 5e) reveals a characteristic peak at ∼860 cm^−^^1^ corresponding to SrCrO_4_. Raman mapping over an 8 × 8 µm region further confirms the localized presence of this SrCrO_4_ phase, providing direct evidence of Cr species deposition. In contrast, the LNC–infiltrated LNC–LNO air electrode exhibits no observable microstructural changes on either the top surface (Figure 5f) or in cross‐section (Figure S12) after long‐term operation. No elemental segregation is detected, and Cr is absent in both surface and cross‐sectional EDS analyses (Figure 5f,g). Consistently, Raman spectra show no signatures of Cr‐related phases, and Raman mapping confirms the absence of SrCrO_4_ or other Cr‐containing compounds (Figure 5h). These results demonstrate that Cr poisoning is effectively suppressed in the LNC–infiltrated LNC–LNO air electrode. For the LSC air electrode, the dominant degradation mechanism originates from the reaction between Sr segregated from the LSC lattice and Cr species evaporated from the metallic interconnect, leading to the formation of insulating Sr–Cr compounds on the electrode surface. The Sr‐free LNC–infiltrated LNC–LNO air electrode is immune to this degradation mechanism owing to the absence of Sr: with no Sr available to react, the incoming Cr species cannot form a stable Sr–Cr compound and are instead carried away with the gas stream, resulting in superior long‐term stability under Cr‐rich operating conditions. Another important contributor to the high stability of the Sr‐free air electrode is the prevention of interfacial degradation. Whereas the formation of SrZrO_3_ at the interlayer/electrolyte interface increases the interfacial resistance and induces a thermal expansion mismatch that causes local mechanical degradation, the Sr‐free air electrode eliminates this interfacial degradation pathway, thereby leading to improved long‐term stability. Finally, the stability of the infiltrated LNC nanoparticles was confirmed by post‐mortem SEM analysis (Figure S13). The nanoparticles retained a size of approximately 50 nm, comparable to their initial size before the durability test, and remained uniformly distributed over the electrode surface without noticeable coarsening or severe agglomeration.

## 3 Conclusion

Although Sr has long been recognized as a primary origin of air electrode degradation, eliminating Sr has remained challenging because it plays a key role in providing the electrical conductivity and catalytic activity required for high‐performance electrodes. In this work, we demonstrate that high electrochemical performance can be achieved using entirely Sr‐free materials by independently engineering electronic/ionic transport and surface catalytic functionality. Notably, the resulting Sr‐free electrode exhibits dramatically improved durability, completely suppressing Cr‐induced degradation even under harsh conditions. Given the rapidly growing demand for high‐temperature electrochemical systems for clean hydrogen production and reliable on‐site power generation, including AI data centers and high‐performance computing infrastructures, operational reliability has become a central requirement for practical deployment. The design strategy presented here provides a scalable and broadly applicable approach for achieving both high performance and exceptional durability, offering a viable pathway toward the commercialization of next‐generation solid oxide electrochemical devices.

## 4 Experimental

### 4.1 Material Synthesis and Characterization

LNO powder was synthesized via the glycine–nitrate process (GNP). Stoichiometric amounts of La(NO_3_)_3_·6H_2_O (Daejung) and Ni(NO_3_)_2_·6H_2_O (Daejung) were dissolved in distilled water, followed by the addition of glycine (Daejung) as a combustion fuel. The glycine‐to‐nitrate molar ratio was fixed at 0.55. The solution was heated on a hot plate until self‐combustion occurred, and the as‐combusted powder was ball‐milled in ethanol, dried at 80°C, sieved, and subsequently calcined at 950°C to obtain phase‐pure LNO.

Commercial La(Ni,Co)O_3_ (LNC, Fuel Cell Materials) and Gd‐doped ceria (GDC, Rhodia) powders were used as received. The crystal structure and phase purity of all powders were analyzed by x‐ray diffraction (XRD, D8 Advance, Bruker) using Cu Kα radiation. Chemical compatibility between constituent materials was evaluated by mixing powders at a 50:50 weight ratio, heat‐treating the mixtures at 950°C, and examining the phase evolution by XRD.

The electrical conductivity of LNO and LNC was measured using a DC four‐probe method. Pellets were fabricated by uniaxially pressing the powders at 150 MPa, followed by sintering at 1300°C. The sintered samples were cut into rectangular bars with dimensions of 16 mm × 1.7 mm × 1.7 mm. Platinum wires were used as current and voltage leads, and conductivity was measured in the temperature range of 350°C–750°C.

### 4.2 Fabrication and Characterization of Symmetric Cells

Symmetric cells were fabricated using 3 mm‐thick GDC discs as substrates. GDC powders were first uniaxially pressed at 150 MPa, followed by cold isostatic pressing at 200 MPa and sintering at 1250°C to obtain dense electrolyte substrates. The paste for the air electrode current‐collecting layer was prepared by mixing LNC powder with a dispersant (Hypermer KD‐6), plasticizer (dibutyl phthalate), binder (BH‐3), and solvent (α‐terpineol) using a planetary mill. The functional‐layer paste was prepared in the same manner using a mixture of 50 wt.% LNC and 50 wt.% LNO powders. These functional and current‐collecting layers were sequentially screen‐printed on both sides of the GDC substrates and sintered at 950°C.

The precursor solution for LNC nanocatalyst infiltration was prepared by dissolving stoichiometric amounts of La(NO_3_)_3_·6H_2_O (Daejung), Ni(OCOCH_3_)_2_·4H_2_O (Daejung), and Co(NO_3_)_2_·6H_2_O (Sigma–Aldrich) in a mixed solvent of distilled water and ethanol. Glycine was added at a glycine‐to‐cation molar ratio of 1:1, followed by urea (Sigma–Aldrich) at a urea‐to‐cation molar ratio of 10:1. All precursor solutions were prepared at a concentration of 0.5 mol L^−^^1^. The solution was infiltrated into the air electrodes and calcined at 650°C to form LNC nanocatalysts.

The microstructures of the fabricated cells were characterized using SEM (Regulus 8230, Hitachi), EPMA (JXA‐8500F, JEOL) and TEM (Talos F200X, Thermo Fisher Scientific). Electrochemical measurements were carried out in an alumina tube reactor at 650°C–750°C under ambient air using a frequency‐response analyzer and potentiostat (Solartron 1260/1287). Electrochemical impedance spectra were analyzed using equivalent circuit modeling and the DRT method. Data fitting was performed with Z‐View 3.1C software, and DRT analysis was conducted using the Z‐ASSIST program (Toyo Corporation). For the DRT analysis, the inductive contribution was determined by a preliminary equivalent‐circuit fit using a sufficiently flexible set of R–CPE elements to accurately reproduce the capacitive arcs. The resulting inductance was then subtracted from the measured spectra, and the corrected spectra were used for the DRT analysis. The regularization parameter was determined using the L‐curve criterion (Figure S14), as described in detail in our previous work [70].

### 4.3 Fabrication and Characterization of Full Cells

Full cells were fabricated using commercial fuel‐electrode‐supported cells (Elcogen), consisting of a ∼400 µm‐thick Ni–YSZ support, a ∼10 µm‐thick Ni–YSZ functional layer, a ∼3 µm‐thick YSZ electrolyte, and a ∼2 µm‐thick GDC interlayer. Air electrodes with an active area of 1.0 cm^2^ were fabricated by screen printing. Four types of air electrodes—single‐phase LNC, LNC–LNO composite, LNC‐infiltrated LNC–LNO, and conventional LSC—were prepared and evaluated. The LSC and LNC–LNO composite electrodes were screen‐printed onto the GDC interlayer and sintered at 950°C. For the LNC–infiltrated LNC–LNO air electrode, LNC nanocatalysts were subsequently infiltrated into the porous backbone following the same procedure used for symmetric cells.

The cells were assembled into single‐cell test fixtures using Crofer 22 APU interconnects, a glass–ceramic sealant, Ni foam, and Pt mesh current collectors. For fuel cell operation, humidified hydrogen (3% H_2_O) and ambient air were supplied to the fuel and air electrodes, respectively. For electrolysis‐mode operation, a gas mixture of 50% H_2_ and 50% H_2_O was supplied to the fuel electrode. Long‐term durability was evaluated at 700°C under constant current densities of 0.5 A cm^−^^2^ in fuel cell mode for 100 h and 1.0 A cm^−^^2^ in electrolysis mode for 200 h. Electrochemical performance was characterized using a frequency‐response analyzer and potentiostat (Solartron 1260/1287) in the temperature range of 650–750°C. After long‐term testing, the microstructure and chemical composition of the electrode surfaces and cross‐sections were analyzed by SEM–EDS. In addition, surface chemical states and secondary phases were examined using Raman spectroscopy (WEVE HEDA).

## 5 Multiphysics Modeling and Overpotential Extraction

To quantify the contribution of the modified air electrode to the overall full‑cell performance, a one‑dimensional (1D) multiphysics model was employed. The model was based on our previously developed 1D [71], isothermal [72], steady‑state framework [55] and a model constructed for the same commercial fuel‑electrode‐supported cell architecture [73].

A reference model representing a commercial full cell with an LSC air electrode was first established and validated against experimental data. Using this validated model, the total overpotential—defined as the difference between the open‑circuit voltage and the cell voltage—was decomposed into the contributions from the fuel electrode (η_*FE*_), air electrode (η_*AE*_), and electrolyte (η_*EL*_), as expressed in Equation (2).

(2) $$E_{OCV}-\;V_{cell}=\eta_{total}=\eta_{FE}+\eta_{AE}+\eta_{EL}$$

The detailed formulations and the corresponding values are provided in Table S4 and Figure S15d, respectively.

All full cells investigated in this study employed the same commercial fuel‑electrode‑supported substrates as the reference cell. Consequently, the fuel‑electrode microstructure and electrolyte thickness were identical for all cells. Therefore, the fuel‑electrode and electrolyte overpotentials in the three Sr‑free air electrodes (LNC, LNC–LNO composite, and LNC–infiltrated LNC–LNO) were assumed to be identical to those of the reference cell. For Sr‐containing air electrodes, secondary phases may form at the electrode/electrolyte interface [74]; in the present model, the additional resistance associated with such interfacial reactions is incorporated into the air electrode overpotential. Based on this assumption, the air electrode overpotential of each Sr‑free cell was determined by subtracting the fuel‑electrode and electrolyte overpotentials obtained from the reference model from the total overpotential measured in the full‑cell polarization experiments shown in Figure 4. Because detailed electrochemical kinetic parameters for Sr‐free materials such as LNC and LNO are not yet sufficiently available in the literature, direct multiphysics modeling of each Sr‐free air electrode was not pursued in this study. Instead, the air electrode overpotential was indirectly estimated through the overpotential‐subtraction procedure described above. Although this approach has inherent limitations, it provides a practical and internally consistent strategy for isolating the air electrode contribution in full cells that share the same fuel‐electrode‐supported substrate and differ only in the air electrode composition. The key aspects of the model are summarized here; additional details can be found in our previous publications [55, 73].

### 5.1 Geometry

As illustrated in Figure S15a, a one‑dimensional through‑thickness model was adopted because in‑plane gradients were negligible under the sufficiently high gas‑flow conditions used in the experiments. The computational domain represents the experimental cell configuration and consists of a 400 µm Ni–YSZ fuel electrode support layer (FESL), 10 µm Ni–YSZ fuel electrode functional layer, 3 µm YSZ electrolyte (EL), 2 µm GDC interlayer (IL), and 15 µm LSC air electrode functional layer (AEFL).

### 5.2 Governing Equations and Electrochemistry

Six conservation equations were solved within the multiphysics framework: conservation of electronic charge, conservation of oxygen‑ion charge, species conservation, and mass and momentum conservation for both the fuel and air gases. The governing equations are summarized in Table 1. The fuel stream was modeled as a binary H_2–_ H_2_O mixture, whereas the air side was treated as an O_2–_N_2_ mixture.

**TABLE 1:** Governing equations used in the multiphysics simulation model.

	**Equations**	**Source term**	**Domains**
e^−^ charge	$\frac{\partial }{\partial z}\;\left(-\sigma_{elec}^{eff}\frac{\partial \phi_{elec}}{\partial z}\right)={\dot{Q}}_{elec}$	${\dot{Q}}_{elec}=-S^{eff}i$	FESL, FEFL, AEFL
O^2−^ charge	$\frac{\partial }{\partial z}\left(-\sigma_{ion}^{eff}\frac{\partial \phi_{ion}}{\partial z}\right)={\dot{Q}}_{ion}$	${\dot{Q}}_{ion}=S^{eff}\;i$	FESL, FEFL, AEFL
		${\dot{Q}}_{ion}=0$	EL, IL
Gas species	$\frac{\partial }{\partial z}\left(\rho \omega_{i}u\right)=\frac{\partial }{\partial z}\left(\rho D_{i}^{eff}\frac{\partial \omega_{i}}{\partial z}\right)+\dot{R_{\dot{i}}}$	${\dot{R}}_{H_{2}}=\frac{-M_{H_{2}}S^{eff}i}{2F}$ ${\dot{R}}_{H_{2}O}=\frac{M_{H_{2}O}S^{eff}i}{2F}$	FESL, FEFL
		$\;{\dot{R}}_{O_{2}}=\frac{M_{O_{2}}S^{eff}i}{4F}$	AEFL
Gas mass	$\frac{\partial }{\partial z}\left(\rho u\right)=\dot{W}$	$\;\dot{W\;}=\frac{\left(M_{H_{2}O}-M_{H_{2}}\right)S^{eff}i}{2F}$	FESL, FEFL
		$\;\dot{W\;}=\frac{M_{O_{2}}S^{eff}i}{4F}$	AEFL
Gas momentum	$\;\frac{\partial p}{\partial z}={\vec{F}}_{Da}$	${\vec{F}}_{Da}=-\frac{\mu }{\zeta }u$	FESL, FEFL, AEFL

Material properties, constitutive relations, and microstructural parameters were adopted from our previously established model for the same commercial Elcogen substrate used in the present study [73]. Porous‑media transport was described using volume‑averaged constitutive relations, and Knudsen diffusion was included in the effective gas diffusivity. Both fuel and air gases were treated as ideal gases.

Hydrogen oxidation/reduction and oxygen reduction/oxidation reactions were considered at the fuel and air electrodes, respectively (Equations 3 and 4). Source terms arising from these electrochemical reactions were consistently incorporated into the corresponding conservation equations.

(3) $$H_{2}\left(g\right)+O^{2-}\left(el\right)⇌H_{2}O\left(g\right)+2e^{-}\left(Ed\right)$$

(4) $$0.5O_{2}\left(g\right)⇌O^{2-}\left(el\right)+2e^{-}\left(Ed\right)$$

Reaction kinetics were described using a modified Butler–Volmer formulation, which has been widely adopted in solid oxide cell modeling studies [75, 76], with exchange current density (*i*_0_) and local activation overpotential ($\eta_{act}^{local}$), as expressed in Equations ((5), (6), (7), (8)):

(5) $$i=i_{0}\;\left\{exp\left(\frac{\alpha nF}{RT}\eta_{act}^{local}\right)-exp\left(-\frac{\left(1-\alpha \right)nF}{RT}\eta_{act}^{local}\right)\right\}$$

(6) $$i_{0,ed}=f\left(p_{i}\right)A_{ed}exp\left(\frac{-E_{a,ed}}{RT}\right)$$

(7) $$\eta_{act}^{local}=\Delta \phi -\Delta \phi^{eq}$$

(8) $$\Delta \phi =\phi_{elec}\;-\phi_{ion}$$

The electrochemical kinetic parameters were primarily adopted from previous literature to minimize arbitrary parameter fitting. For the fuel electrode, the kinetic parameters were taken from our previous study [71], which considered the same commercial Elcogen substrate and calibrated and validated the parameters against experimental data over a wide range of fuel compositions (from 3% H_2_O + 97%H_2_ to 80% H_2_O + 20%H_2_) and operating temperatures (600–750°C), including the conditions used in the present study. For the air electrode, the pre‐exponential factor of the exchange current density (*A_AE_*) was the only fitted parameter used to reproduce the experimental full‐cell performance, while the remaining parameters were adopted from a previous study on LSC(F) air electrodes [71, 73, 77]. The resulting parameter values are summarized in Table S5.

### 5.3 Boundary and Operating Conditions

Boundary conditions were applied at the bottom surface of the fuel‑electrode support layer (*z*  =  0) and the top surface of the air electrode functional layer (*z*  = *t_cell_*), consistent with the experimental operating conditions (Table 2). Electrical ground and current density conditions were imposed at the bottom and top boundaries, respectively. The applied current density corresponded directly to the experimental values. For species conservation, constant inlet gas compositions were specified as mass fractions corresponding to the supplied values, as concentration gradients in the gas channels are negligible under the high gas‑flow (over‑supplied) experimental conditions. Accordingly, the fuel molar composition was set to 97% H_2_ + 3% H_2_O in fuel cell mode (*j_op_* > 0) and 50% H_2_ + 50% H_2_O in electrolysis mode (*j_op_* < 0). The air molar composition was fixed at 21% O_2_ + 79% N_2_ for all operating modes. Atmospheric pressure was imposed for the gas mass and momentum conservation equations.

**TABLE 2:** Boundary and operating conditions used in the multiphysics simulation model.

Boundary	Charge	Species	Mass and momentum
*z* = 0	$\phi_{elec}=\phi_{gd}$	$\;\omega_{H_{2}}=\omega_{H_{2},in}$	*p* = *p_atm_*
*z* = *t_cell_*	$-\sigma_{elec}^{eff}\;\frac{\partial \phi_{elec}}{\partial z}=j_{op}$	$\;\omega_{O_{2}}=\omega_{O_{2},in}$	*p* = *p_atm_*

### 5.4 Numerical Implementation and Experimental Validation

The model was implemented in COMSOL Multiphysics v6.1 [78]. All governing equations were discretized using the finite element method (FEM). To capture steep gradients near the electrolyte interfaces, a non‑uniform mesh with local refinement in the electrolyte and adjacent electrode regions was employed (Figure S15a). All equations were solved simultaneously using a fully coupled approach. The nonlinear system was solved using the Newton method, and the linearized system was handled using the PARDISO direct solver. Convergence was achieved when the relative residual of all governing equations fell below 10^−4^.

Model validity was assessed by comparison with experimentally measured full cell I–V curves (Figure S15b). The simulated results showed good agreement with the experimental curves measured at 650°C, 700°C, and 750°C, confirming that the governing equations, material properties, and reaction kinetics were appropriately selected. A mesh‑independence test was also conducted to ensure numerical stability. As shown in Figure S15c, the predicted electrochemical performance became insensitive to further mesh refinement (twice the normal mesh density), confirming grid‑independent solutions.

## Nomenclature

*E_a_*
: Activation energy (kJ mol–1)
*V_cell_*
: Cell voltage (V)
α: Charge transfer coefficient (–)
*i*
: Charge transfer current density (A m–2)
μ: Dynamic viscosity (Pa s)
*D_i_*
: Diffusion coefficient of species i (m2 s–1)
σ: Electrical conductivity (S m–1)
ϕ
: Electric potential (V)
*i*_0_
: Exchange current density (A m–2)
*F*
: Faraday constant (C mol–1)
*R*
: Gas constant (J mol–1 K–1)
ζ: Gas permeability (m2)
ω_*i*_
: Mass fraction of species i (–)
*M_i_*
: Molecular weight of species i (kg mol–1)
*n*
: Normal outward unit vector (–)
η: Overpotential (V)
*A*
: Pre‐exponential factor (A m–2)
*p*
: Pressure (Pa)
*S*
: Reaction site per unit volume (m2 m–3)
${\dot{R}}_{i}$
: Source of gas species i (kg m–3)
$\dot{W}$
: Source of mass (kg m–3)
$\vec{u}$
: Velocity (m s–1)

### Acronyms

    

CC Current collector
EL Electrolyte
Ed Electrode
FESL Fuel electrode supporting layer
FEFL Fuel electrode functional layer
IL Interlayer
Kn Knudsen
AEFL air electrode functional layer
TPB Triple‐phase boundary

### Subscripts

    

act activation
ct charge transfer
elec electronic
FE fuel electrode
ion ionic
AE air electrode
ohm ohmic

### Superscripts

    

eff effective
eq equilibrium
0 standard

## Author Contributions

J.‐eun Won: Methodology, Investigation, Writing – original draft, W. Lee: Software, J. Seo: Investigation, D. Yoon: Investigation, H.‐I. Ji: Investigation, H. Kang: Formal Analysis, J. H. Kim: Formal Analysis, H. Kim: Methodology, J.W. Kim: Methodology, J. Hong: Supervision, Writing – review & Editing, K. J. Yoon: Conceptualization, Supervision, Writing – review & Editing.

## Conflicts of Interest

The authors declare no conflicts of interest.

## Supporting information




**Supporting File**: advs77023‐sup‐0001‐SuppMat.docx.

## Data Availability

The data that support the findings of this study are available in the supplementary material of this article.
