# Supplementary material for: Decoupling Electronic–Ionic Transport and Catalysis Enables High‐Performance, Chemically Stable Sr‐Free Air Electrodes for High‐Temperature Solid Oxide Cells
Source: Adv Sci (Weinh). 2026 Aug 5;13(60):e77023. doi: 10.1002/advs.77023 (PMC13440377; doi:10.1002/advs.77023)
Supplement: Supplementary file 1 [file ADVS-13-e77023-s001.docx]

**Supporting Information**

***Decoupling Electronic–Ionic Transport and Catalysis Enables High-Performance, Chemically Stable Sr-Free Air Electrodes for High-Temperature Solid Oxide Cells***

*Ji-eun Won, Wooseok Lee, Jaehyun Seo, Dogeun Yoon, Ho-Il Ji, HeeChan Kang, Jun Hyuk Kim, Hwitae Kim, Ji Wan Kim, Jongsup Hong^*^, Kyung Joong Yoon^*^*

J.-eun Won, W. Lee, J. Seo, D. Yoon, H.-I. Ji*,* K. J. Yoon

Hydrogen Energy Materials Research Center, Korea Institute of Science and Technology, Seoul, Republic of Korea

E-mail: kjyoon@kist.re.kr

J.-eun Won, D. Yoon, J. Hong

School of Mechanical Engineering, Yonsei University, Seoul 03722, Republic of Korea

E-mail: jongsup.hong@yonsei.ac.kr

H.-I. Ji

Nanomaterials Science and Engineering, Korea University of Science and Technology (UST), KIST Campus, Seoul 02792, Republic of Korea

H. Kang, J. H. Kim

Department of Future Energy Engineering, Sungkyunkwan University, Suwon, Republic of Korea

J. H. Kim

SKKU Institute of Energy Science and Technology (SIEST), Sungkyunkwan University, Suwon, Republic of Korea

H. Kim, J.W. Kim

Materials Research & Engineering Center, Hyundai Motor Company, Uiwang-si, Gyeonggi-do, Republic of Korea

**Figure S1.** Electrical conductivity of LNC and LNO measured at 350-750 °C in air.

**Figure S2.** Cross-sectional SEM image of a symmetric cell with an LNC–LNO composite electrode.

**Figure S3.** Cross-sectional SEM image of a symmetric cell with a single-phase LNC electrode.

**Figure S4.** Impedance spectra of symmetric cells with LNC–LNO composite electrodes having LNC:LNO weight ratios of 60:40, 50:50, and 40:60, measured at 700 °C.

**Figure S5.** *pO_2_*-dependent impedance analysis of the LNC–LNO composite electrode. (a) Nyquist (upper) and Bode (lower) plots of the impedance spectra measured at 700 °C. (b) Series of DRT spectra obtained at different *pO_2_*. (c) Equivalent-circuit fitting of the impedance spectra using a model constructed from the DRT results. (d) Dependence of each *R_p_* component on *pO_2_*, showing the corresponding reaction orders.

**Figure S6.** SEM images of infiltrated (a) La(Ni₀.₇Co₀.₃)O₃-_δ_, (b) La(Ni₀.₆Co₀.₄)O₃-_δ_, and (c) La(Ni₀.₄Co₀.₆)O₃-_δ_ nanoparticles in the LNC–LNO composite electrode.

**Figure S7.** Cross-sectional SEM image of a full cell with a single-phase LNC electrode.

**Figure S8.** Full-cell test assembly shown in sequence: (a) metallic interconnect with channel/rib geometry, (b) current collector, (c) cell mounted on the assembly, and (d) glass-ceramic sealant framing the cell.

**Figure S9.** I–V curves of the LSC air electrode measured in fuel cell and electrolysis modes, with the corresponding power density curves obtained in fuel cell mode.

**Figure S10.** Stability test results for the cells with the LNC-infiltrated LNC–LNO air electrode and the LSC air electrode under fuel-cell-mode operation at a constant current density of 0.5 A cm^-2^ and 700 °C.

**Figure S11.** Cross-sectional SEM image of the surface region of the LSC air electrode after long-term operation.

**Figure S12.** Cross-sectional SEM image of the surface region of the LNC-infiltrated LNC–LNO air electrode after long-term operation.

**Figure S13.** Post-mortem SEM image of the LNC nanocatalysts in the LNC-infiltrated LNC–LNO air electrode after the 200-h long-term durability test.

**Figure S14.** Plot of solution norm versus misfit norm for the LNC–LNO composite electrode measured in air at 700 °C, shown as an example of the L-curve criterion used to determine the regularization parameter in the DRT analysis.

**Figure S15**. One-dimensional multiphysics simulation model and validation results. (a) Model geometry and computational mesh. (b) Model validation by comparison with the experimental I–V curves shown in Figure S9. (c) Grid-independence test results. (d) Decomposition of cell overpotential into fuel-electrode, air-electrode, and electrolyte contributions.

**Table S1.** Peak-area analysis of the O 1s XPS spectra, showing the relative percentage of each deconvoluted component.

| ­­Air electrode | Area of O 1s peaks (%) | | | | |
| --- | --- | --- | --- | --- | --- |
|  | O_H_ | O_ad_ | O_2_^2-^/O^-^ | O_lat_ | |
| LNC | 16.71 | 27.62 | 22.57 | 33.10 |  |
| LNC-LNO | 15.48 | 36.70 | 22.51 | 25.31 |  |

**Table S2.** Comparison of maximum power densities measured in fuel cell mode for cells with selected Sr-free air electrodes reported in the literature [19, 40].

| ­­Air electrode | Maximum Power Density (W cm^-2^) | | | Ref. |
| --- | --- | --- | --- | --- |
|  | **650 °C** | **700 °C** | **750 °C** |  |
| LNC | 0.26 | 0.50 | 0.78 | This work |
| LNC-LNO | 0.32 | 0.65 | 1.19 |  |
| LNC infiltrated LNC-LNO | 0.47 | 0.90 | 1.44 |  |
| LSC | 0.45 | 0.8 | 1.21 |  |
| LCN | - | 0.80 | 1.25 | [19-e] |
| LCaFN | - | - | 0.8 | [19-f] |
| LBF | - | 0.27 | 0.46 | [40-a] |
| HE-LMO | - | 0.26 | 0.42 | [40-b] |
| LBFZO | - | 0.32 | 0.36 | [40-c] |
| LBNFTi | 0.33 | 0.58 | 0.81 | [40-d] |
| LPN | 0.32 | 0.44 | 0.5 | [40-e] |

**Table S3.** Comparison of current densities at the thermoneutral voltage (*V_TN_*) measured in electrolysis mode for cells with selected Sr-free air electrodes reported in the literature [41].

| Air electrode | Current Density (A cm^-2^) @*V_TN_* | | | Ref. |
| --- | --- | --- | --- | --- |
|  | **650 °C** | **700 °C** | **750 °C** |  |
| LNC | 0.08 | 0.22 | 0.46 | This work |
| LNC-LNO | 0.28 | 0.63 | 1.27 |  |
| LNC infiltrated LNC-LNO | 0.51 | 1.01 | 1.69 |  |
| LSC | 0.38 | 0.94 | 1.58 |  |
| BLF | - | 0.85 | 1.11 | [41-a] |
| LNCuO | 0.19 | 0.37 | 0.63 | [41-b] |
| PBNO | - | 0.39 | 0.55 | [41-c] |
| PBCF | - | 0.65 | 1.12 | [41-d] |

**Table S4**. Detailed equations for averaged overpotentials computed from multiphysics simulation model.

| **Overpotential** | **Expression** |
| --- | --- |
| $\eta_{FE}$ | $\eta_{act,FE}+\eta_{conc,FE}+\eta_{ohm,ion,FE}+\eta_{ohm,elec,FE}$ |
| $\eta_{AE}$ | $\eta_{act,AE}+\eta_{conc,AE}+\eta_{ohm,ion,AE}+\eta_{ohm,elec,AE}$ |
| $\eta_{EL}$ | $\eta_{ohm,ion,EL}+\eta_{ohm,ion,IL}$ |
| $\eta_{act,FE}$ | $\iiint_{FESL} S^{eff}i\eta_{act}^{local}dV+\iiint_{FEFL} S^{eff}i\eta_{act}^{local}dV$ |
| $\eta_{conc,FE}$ | $\iiint_{FESL} S^{eff}i\eta_{conc,FE}^{local}dV+\iiint_{FEFL} S^{eff}i\eta_{conc,FE}^{local}dV$  *where,* $\eta_{conc,FE}^{local}={\Delta\phi}_{FE}^{eq}-{\Delta\phi}_{FE,in}^{eq}$ |
| $\eta_{act,AE}$ | $\iiint_{AEFL} S^{eff}i\eta_{act}^{local}dV$ |
| $\eta_{conc,AE}$ | $\iiint_{AEFL} S^{eff}i\eta_{conc,AE}^{local}dV$  *where,* $\eta_{conc, AE}^{local}={\Delta\phi}_{AE}^{eq}-{\Delta\phi}_{AE,in}^{eq}$ |
| $\eta_{ohm,ion}$ | $\iiint_{FESL} \sigma_{ion}^{eff}\left\vert\nabla\phi_{ion} \right\vert^{2}dV+\iiint_{FEFL} \sigma_{ion}^{eff}\left\vert\nabla\phi_{ion} \right\vert^{2}dV+\iiint_{EL} \sigma_{ion}^{eff}\left\vert\nabla\phi_{ion} \right\vert^{2}dV+\iiint_{IL} \sigma_{ion}^{eff}\left\vert\nabla\phi_{ion} \right\vert^{2}dV+\iiint_{AEFL} \sigma_{ion}^{eff}\left\vert\nabla\phi_{ion} \right\vert^{2}dV$ |
| $\eta_{ohm,elec}$ | $\iiint_{FESL} \sigma_{elec}^{eff}\left\vert\nabla\phi_{elec} \right\vert^{2}dV+\iiint_{FEFL} \sigma_{elec}^{eff}\left\vert\nabla\phi_{elec} \right\vert^{2}dV+\iiint_{AEFL} \sigma_{elec}^{eff}\left\vert\nabla\phi_{elec} \right\vert^{2}dV$ |

**Table S5**. Electrochemical reaction kinetic parameters used in the multiphysics simulation model.

| **Reaction site** | **Parameter** | **Value or expression** | **Unit** | **Reference** |
| --- | --- | --- | --- | --- |
| Ni/YSZ Fuel  electrode | $A$ | 2.37×10^11^ | A m^-2^ | [46] |
|  | $E_{a}$ | 105 | kJ mol^-1^ |  |
|  | $f(p_{i})$ | $\frac{\left( {p_{H_{2}}}/{p_{H_{2}}^{*}} \right)^{\frac{1}{4}} \left( {p_{H_{2}O}}/{p^{\circ}} \right)^{\frac{3}{4}}}{1+\left( {p_{H_{2}}}/{p_{H_{2}}^{*}} \right)^{\frac{1}{2}}}$ | - |  |
|  | $n$ | 2 | - |  |
|  | $\alpha$ | 0.75 | - |  |
| LSC Air  electrode | $A$ | 6.735×10^8^ | A m^-2^ | fitted |
|  | $E_{a}$ | 120 | kJ mol^-1^ | [46] |
|  | $f(p_{i})$ | $\frac{\left( {p_{O_{2}}}/{p_{O_{2}}^{*}} \right)^{\frac{1}{4}}}{1+\left( {p_{O_{2}}}/{p_{O_{2}}^{*}} \right)^{\frac{1}{2}}}$ | - |  |
|  | $n$ | 2 | - |  |
|  | $\alpha$ | 0.75 | - |  |
